# Volumetric FDG-PET predicts overall and progression- free survival after 14 days of targeted therapy in metastatic renal cell carcinoma

**DOI:** 10.1186/1471-2407-14-408

**Published:** 2014-06-06

**Authors:** Jacob Farnebo, Per Grybäck, Ulrika Harmenberg, Anna Laurell, Peter Wersäll, Lennart K Blomqvist, Anders Ullén, Per Sandström

**Affiliations:** 1 Department of Diagnostic Radiology, Karolinska University Hospital and Department of Molecular Medicine and Surgery, Karolinska Institutet, Stockholm, Sweden; 2 Department of Oncology, Karolinska University Hospital and Department of Oncology and Pathology, Karolinska Institutet, Stockholm, Sweden; 3 Department of Oncology, Academic Hospital, Uppsala, Sweden

**Keywords:** FDG-PET, Renal cell carcinoma, Biomarker, Targeted therapy, Total lesion glycolysis

## Abstract

**Background:**

To determine whether changes in the metabolism of metastatic renal cell carcinoma (mRCC) assessed by F18-FDG-PET after 14 and 28 days of treatment with tyrosine kinase inhibitors can predict overall and progression- free patient survival.

**Methods:**

Thirty-nine consecutive patients with mRCC were included prospectively and underwent PET examinations prior to and after 14 and 28 days of standard treatment with sunitinib (n = 18), sorafenib (n = 19) or pazopanib (n = 2). The PET response was analyzed in terms of SUVmax, SULpeak, and total lesion glycolysis and a positive response (defined as a 30% reduction) compared to overall and progression- free survival.

**Results:**

Thirty-five patients with at least one metabolically active metastatic lesion prior to treatment underwent additional FDG-PET examinations after 14 (n = 32) and/or 28 days (n = 30) of treatment. Changes in either SULpeak or total lesion glycolysis were correlated to both progression-free and overall survival (for TLG2.5 responders, HR = 0.38 (95% CI: 0.18-0.83) and 0.22 (95% CI: 0.09-0.53), and for TLG50 responders, HR = 0.25 (0.10-0.62) and 0.25 (95% CI: 0.11-0.57) and for SULpeak responders, HR = 0.39 (95% CI: 0.17-0.91) and 0.38 (95% CI: 0.15-0.93), respectively). In contrast SUVmax response did not predict progression- free or overall survival (HR = 0.43 (95% CI: 0.18-1.01) and 0.50 (95% CI: 0.21-1.19), respectively).

**Conclusions:**

Assessment of early changes in SULpeak and total lesion glycolysis undergoing treatment with tyrosine kinase inhibitors by FDG-PET can possibly predict progression- free and overall survival in patients with mRCC.

## Background

In the last decade, new antiangiogenic therapies such as the tyrosine kinase inhibitors (TKIs) sunitinib, sorafenib and pazopanib [1-3] have changed the management of patients with metastatic renal cell carcinoma (mRCC). Eventually all patients experience relapse and the duration of the drug response varies widely with certain patients receiving little benefit. Traditional assessment of drug response with computed tomography has limitations in the case of mRCC, since metastases often enter a period of dormancy and tumor shrinkage occurs only after a cascade of cellular and subcellular changes [4]. Thus, novel biomarkers of response are required to allow early consideration of alternative treatment for non-responders as well as to reduce unnecessary side-effects and costs.

Positron emission tomography (PET) employing 18 F-flouro-deoxyglucose (FDG) allows detection and staging of many cancers, revealing early changes in tumor metabolism that might be valuable biomarkers for drug response [5]. A recent investigation using this technique before and after a one-month treatment successfully predicted progression-free survival (PFS) in patients with mRCC [6], but a similar study could only predict overall survival (OS) [7] after 4 months treatment. In both cases the maximal standardized uptake (SUVmax) was the sole FDG-PET parameter utilized as an indicator of metabolism. Although SUVmax, the highest uptake of FDG in one voxel (image volume) of the tumor, is indeed most often used in clinical practice, several other PET-parameters are being explored [8]; including metabolic tumor volume (MTV), total lesion glycolysis (TLG) and peak standardized uptake normalized to lean body mass (SULpeak).

Here, the hypothesis that alterations in the uptake of FDG by mRCC after only 14 days of treatment correlates both with progression-free and overall survival was tested. We also predicted that the manner in which this uptake is measured plays a critical role in assessment of the metabolic response.

## Methods

Thirty-nine selected patients with metastatic renal cell carcinoma who were scheduled to start treatment with sorafenib, sunitinib or pazopanib at the Karolinska University Hospital (Stockholm, Sweden) or Uppsala University Hospital (Uppsala, Sweden) between April 2006 and December 2010 agreed to participate in this study. Written informed consent was obtained from all patients. Their baseline characteristics are documented in Table 1. Approval was obtained from the Stockholm Regional Ethical Review Board (2007/1551-31/3).

**Table 1:** The baseline characteristics of the 39 participants

**Mean age (years)**	**65**
Histology (clear cell/papillary)	38/1
Prognostic risk	
MSKCC (low/intermediate/high)	8/24/4
Heng (low/intermediate/high)	7/21/8
ECOG performance status (0-1/>1)	33/6
Treatment with	
sorafenib/sunitinib/pazopanib	19/18/2
Nephrectomy (y/n)	37/2
Prior treatment	
None	20
Interferon-alpha	7
sunitinib	11
Chemotherapy	1

### Treatment

Following a baseline PET scan, 18 patients were treated with sunitinib, 19 with sorafenib and two with pazopanib. 16 of those in the sunitinib group had had no prior treatment while one patient had already received interferon-alpha and one other had received gemcitabine. Among those treated with sorafenib two had had no prior treatment, while 11 received sunitinib, 5 interferon-alpha and one both interferon-alpha and sunitinib. Neither patient administered pazopanib had received prior treatment. One patient entered the study twice, initially receiving sunitinib and later sorafenib. All treatment was administered in accordance with the recommendations: in the case of sunitinib a starting dose of 50 mg once daily for four week periods separated by two weeks off treatment; for those receiving sorafenib, a starting dose of 400 mg twice daily; and for pazopanib a dose of 800 mg once daily.

Decisions concerning treatment were based on standard anatomic assessment of response by CT and evaluated according to RECIST1.1 [9]. The PET assessments did not influence these decisions but the treating physician was not blinded to the PET results.

### PET examinations

PET examinations were carried out (immediately prior to and after 14 and 28 days of treatment) using the standard clinical protocol. The first 5 patients (included during 2006 and 2007) were examined with a ECAT EXACT 31 PET camera (CTI, Knoxville, Tenn., USA) and 30 of the subsequent patients with a Biograph 64 Truepoint PET/CT (Siemens Medical Solutions, Erlangen Germany) scanner (during 2008–2011). In the case of two patients from Uppsala University Hospital Discovery ST PET/CT scanner (GE Healthcare) was employed. For each patient all three scans were performed on the same machine.

One hour after intravenous injection of 4 MBq FDG/kg the patients were scanned from the base of their skulls to the proximal aspects of the thighs. They were instructed to fast for at least 6 hours prior to examination and the blood level of glucose was measured routinely. In addition, a low-dose attenuation correction and a full-dose diagnostic CT were performed. Contrast medium was injected intravenously in connection with the baseline and third scan. Assessment was acheived with Siemens True-D Syngo software.

### Image analysis

All PET scans were analyzed retrospectively by the same radiologist (JF or PG), who had received no information concerning either the clinical or radiological outcome. Metastatic lesions were identified by correlating focal uptake of FDG with the corresponding CT images, with correction for normal physiological uptake. A two-dimensional circle drawn around the metastatic lesion in the transverse plane allowed the software to plot a three-dimensional metabolic volume. Manual adjustment in the cranio-caudal plane was occasionally required to exclude uptake by normal proximate tissue. For semi-quantitative analysis of FDG uptake, the SUVmax was identified. The metabolic tumor volume (MTV) was defined as an isocontour along either 50% of SUVmax or a fixed SUV threshold of 2.5 within a three-dimensional region of interest (ROI). For assessment of total lesion glycolysis (TLG), the average SUV within the tumor lesion was multiplied by its MTV to obtain TLG50 and TLG2.5, respectively.

For assessment according to PERCIST1.0 [10], all SUV values were normalized to lean body mass (SUL). A sphere ROI with a volume of one cubic centimeter was drawn around the region of the tumor demonstrating most rapid uptake of FDG and the average uptake within that volume defined as SULpeak.

### Assessment of the metabolic response

For comparison, the metabolic response was assessed in different ways. Metastatic lesions that avidly took up FDG were identified from the baseline scan and this uptake evaluated on the basis of SUVmax, SULpeak, TLG50 and TLG2.5. No more than five lesions in total and two lesions per organ were examined in each patient. The sum of the uptake by these target lesions at baseline and the percentage change after 14 and 28 days of treatment were calculated. In a parallel analysis, the lesion that took up FDG the most rapidly (hottest lesion) at baseline was evaluated on its own.

Metabolic response was defined as a 30% reduction in either SUVmax, TLG50 or TLG2.5. Metabolic progression was defined as the appearance of new metabolic active lesions typical this type of cancer and/or at least a 30% increase in SUVmax, TLG50 or TLG2.5 in comparison to baseline. When neither progression nor regression was observed, the tumor was considered to be metabolically stable.

For assessment according to the PERCIST 1.0 criteria [10], a greater reduction than 30% in SULpeak was defined as partial metabolic response (PMR). Progressive metabolic disease (PMD) was defined as the appearance of new metabolic lesions or an increase in the SULpeak of more than 30% and/or in TLG50 of more than 75%. Once again, the SULpeak of both the hottest lesion alone and of all target lesions combined were analyzed.

### Assessment of the clinical outcome

The patients were followed-up 3, 5, 7 and 9 months after initiation of treatment or earlier if clinically indicated. Assessment of the anatomic response with CT was carried out using RECIST 1.1 [9]. The time to progression was calculated as the period from date of the baseline scan to the detection of progressive disease by CT. Overall survival was calculated from the date of the baseline scan to death or to the date of the final follow-up (patients still alive are displayed as censored cases in the Kaplan-Meier survival graphs).

### Statistical analyses

Overall survival and progression-free survival were analyzed with the Kaplan-Meier procedure, and the log-rank test applied for statistical comparison of independent subgroups. Univariate Cox proportional hazard analysis with 95% confidence intervals was employed to evaluate the impact of baseline characteristics on overall survival (Table 2) as well as the association between metabolic PET response and overall survival. The difference between pre- and post therapeutic measurements was calculated as continuous variables and by percentage change. Hazard ratios were provided. Statistical analyses were conducted using the IBM SPSS Statistics software (version 21.0).

**Table 2:** Univariate analysis of clinical parameters observed in connection with the baseline FDG-PET that were associatied with overall survival

	**HR (95% CI)**
The hottest lesion: high SUVmax^a^	3.56(1.63-7.76)*
The hottest lesion: high SULpeak^a^	2.67(1.22-5.84)*
The hottest lesion: high TLG50^a^	2.45(1.14-5.27)*
The hottest lesion: high TLG2.5^a^	1.74(0.83-3.63)
Rating of Heng factor: 1 versus 2 and 3	0.33(0.11-0.96)*
ECOG performance status: 0 versus 1 and 2	1.94(0.91-4.14)
Pretreatment: yes versus none	1.57(0.75-3.27)

^a^Comparing above and below median value.

* = Statiscially significant.

## Results

### Patient outcome

Of the 39 patients who underwent a FDG-PET scan prior to treatment, four patients exhibited no metabolically active lesions and were therefore excluded from further evaluation. Four had lesions that avidly took up FDG but did not fulfill the PERCIST1.0 criteria for measurable target lesions. Four remained alive at the time of the final follow-up (September 2012) and all the others had died from metastatic disease. The median progression-free and overall survival was 159 days (range 14–1153 days) and 652 days (range 42–2310 days), respectively. The median survival of the censored patients was 1629 days (range 1280–2683) and the reverse Kaplan-Meier estimate of the median follow-up 1467 days (95% CI: 966–1967 days).

### Results of the baseline PET scan

The median time between the baseline PET examination and initiation of treatment was 2.5 days (range 0–14). The average number of tumor lesions avidly taking up FDG at baseline was 2.3 (median 2, range 1–5) and for the hottest lesion, SUVmax ranged from 2.6-25.3 (median 7.1), SULpeak from 2.1-16.1 (median 4.6), TLG50 from 6–1128 (median 58) and TLG2.5 from 3–5511 (median 115). Univariate analysis of the hottest lesion revealed that SUVmax, SULpeak and TLG50 values above the median were significantly correlated both with shorter PFS (not shown) and OS (Table 2). Heng score (good versus poor) at baseline was associated with overall survival but there was no association between ECOG status or previous treatment and overall survival (Table 2).

### Metabolic response following 14 days of treatment

**Figure 1:**
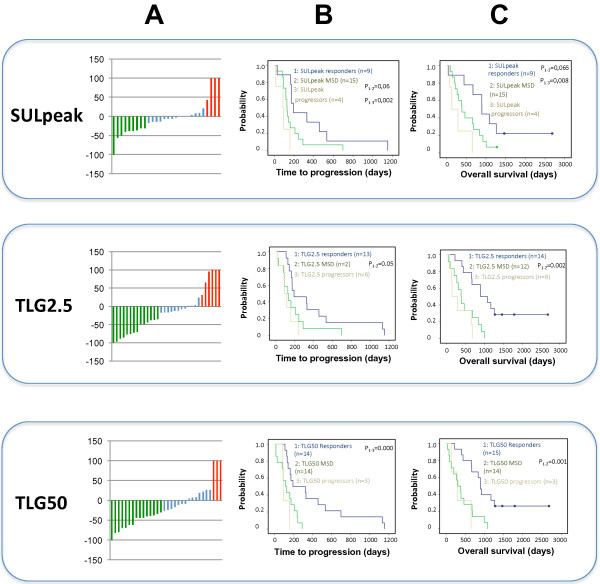
**The metabolic response of patients with mRCC after 14 days treatment with tyrosine kinase inhibitors. (A)** Waterfall plots of the metabolic response of patients with mRCC after 14 days of treatment with tyrosine kinase inhibitors as reflected in SULpeak, TLG75 and TLG50. Kaplan-meier graphs comparing responders and non-responders with regards to time to progression **(B)** and overall survival **(C)**.

**Figure 2:**
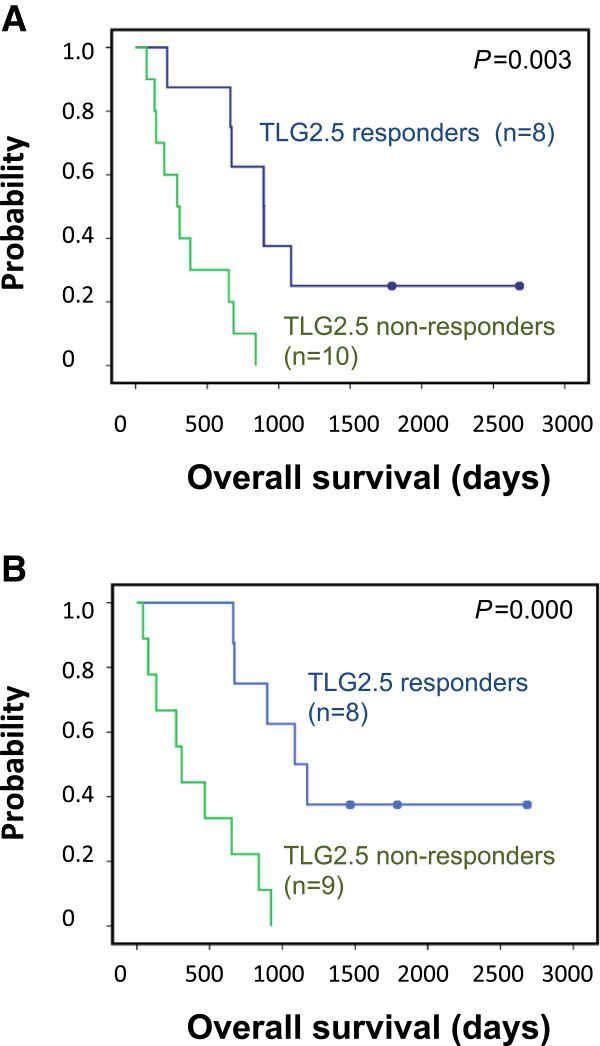
**Kaplan-Meier survival curves comparing subgroups of patients. (A)** Comparing the subgroup of patients with an intermediate prognostic Heng factor score and **(B)** the group of patients with no previous treatment, on the basis of metabolic response or lack of metabolic response (metabolic stable disease and metabolic progress) to 14 days of treatment with tyrosine kinase inhibitors as reflected in TLG2.5.

Thirty-two patients underwent a PET scan after a median of 14 days (range 10–17) of treatment, with determination of SULpeak in 28 and of TLG50 and TLG2.5 in all 32 participants. Metabolic response and progress after 14 days of treatment are displayed in Tables 3, 4 and Figure 1. On the basis of total lesion glycolysis as reflected in TLG50 and TLG2.5, more of the patients were responders or demonstrated metabolic progress than indicated by the SUVmax. Metabolic response indicated by SULpeak, TLG50 and/or TLG2.5 was significantly associated to overall survival and PFS (Figures 1, 2 and Table 4), while there was no significant association between the SUVmax response and overall survival (Table 4). In analyses of the hottest lesions SULpeak TLG2.5 and TLG50 demonstrated significant associations (Table 4).

**Table 3:** The number of patients demonstrating a metabolic response following 14 and 28 days of treatment

	**After 14 days**		**After 28 days**	
	**Metabolic response**	**Metabolic progress**	**Metabolic response**	**Metabolic progress**
SULpeak^a^	9/28	8/28	11/28	4/28
SUVmax	8/32	5/32	12/30	6/30
TLG2.5	14/32	6/32	14/30	6/30
TLG50	15/32	3/32	12/30	5/30
HottestSULpeak^a^	9/28	7/28	10/28	5/28
HottestSUVmax	8/32	4/32	8/30	4/30
HottestTLG2.5	14/32	6/32	14/30	6/30
HottestTLG50	17/32	5/32	15/30	6/30

^a^Assessed according to PERCIST1.0. Only 28 patients had a lesion that fulfilled the PERCIST criteria.

**Table 4:** Univariate Cox regression analysis of the parameters of metabolic response predictive of overall survival

	**After 14 days**		**After 28 days**	
	Above versus below the median value*	**Metabolic response versus no response**	Above versus below the median value*	**Metabolic response versus no response**
SULpeak^a^	2.77(1.10-6.98)	0.38(0.15-0.93)*	4.07(1.54-10.78)*	0.94(0.41-2.20)
SUVmax	1.31(0.62-2.74)	0.58(0.26-1.28)	2.06(0.93-4.54)	0.50(0.21-1.19)
TLG2.5	5.35(2.08-13.75)*	0.22(0.09-0.53)*	3.54(1.52-8.27)*	0.40(0.18-0.92)*
TLG50	4.07(1.77-9.38)*	0.25(0.11-0.57)*	4.41(1.79-10.87)*	0.30(0.12-0.74)*
HottestSULpeak^a^	1.61(0.71-3.66)	0.38(0.15-0.93)*	3.20(1.25-8.18)*	0.84(0.37-1.91)
HottestSUVmax	1.32(0.60-2.93)	0.60(0.25-1.44)	1.92(0.88-4.18)	0.97 (0.41-2.31)
HottestTLG2.5	6.15(2.32-16.32)*	0.29(0.12-0.66)*	5.43(2.17-13.58)*	0.18(0.07-0.44)*
HottestTLG50	2.30(1.06-4.99)*	0.20(0.09-0.47)*	2.65(1.15-6.08)*	0.35(0.15-0.79)*

^a^According to PERCIST1.0, * = Significant within a confidence interval of 95%.

Analysis of the intermediate group according to the Heng classification [11] revealed that patients whose TLG2.5 did not respond had a significantly poorer prognosis than responders (Figure 2A). In a separate analysis of patients who had received no pre-treatment, metabolic response was significantly associated with overall survival (Figure 2B).

### Metabolic response following 28 days of treatment

Thirty patients conducted a PET-scan after 28 days of treatment (median 27, range 20–76). Three patients conducted only a baseline and a 28 day PET and five patients conducted only a baseline and a 14 day PET. Metabolic response and progress after 28 days of treatment are displayed in Tables 3, 4 and Figure 1.

## Discussion

To our knowledge this is the first demonstration that FDG-PET can be used to predict survival in patients with metabolic active metastatic renal cell cancer after only 14 days of treatment with a tyrosine kinase inhibitor. Previous reports have either failed to predict outcome [7] or have involved evaluation at later timepoints [6,12,13]. Present findings highlight the value of volume-based metabolic parameters (such as SULpeak and TLG) in assessing the response of patients with mRCC by FDG-PET.

In line with previous reports (5, 6), SUVmax failed to predict outcome after 14 and 28 days of treatment. A possible explanation could be that SUVmax only reflects a single voxel subjected to a highly variable degree of noise [14], and is thus less reliable for detecting subtle metabolic changes. Recently, SULpeak has been recommended as a more robust alternative. Indeed we found here that the response in SULpeak was more closely correlated to clinical outcome than the change in SUVmax. One problem associated with the use of SULpeak is how to define the region of interest, the choice of which can influence the value obtained substantially [15]. The volumetric thresholds of SUV 2.5 and 50% of SUVmax were selected here after an initial analysis of several different fixed thresholds (41%, 50%, 75% and 90% of SUVmax). Use of a too low threshold sometimes resulted in too much background interference and unrealistic large tumor volume. On the other hand a too high threshold led to an unnecessary reduction of the metabolic volume of the tumor. We observed a significant correlation between either the MTV or TLG response and clinical outcome, with TLG appearing to be more compelling, since this parameter contains more information about the of FDG-uptake. Comparison of analyses of the hottest lesion and multiple lesions revealed that the association with clinical outcome was stronger when several lesions were analyzed.

Among several studies of monitoring treatment with FDG-PET published during the past 20 years, a consistent finding has been that this approach allows more accurate differentiation between treatment induced fibrosis/necrosis and viable tumor tissue. In malignant lymphoma [16,17], FDG-PET now plays a central role in defining tumor response. In patients whose gastrointestinal stromal tumors were treated with imatinib, the FDG-PET response after only 1 week of treatment (which is much sooner than anatomic changes are expected) proved to be a valuable predictor of long-term outcome [18]. In addition, contrast-enhanced ultrasound was able to detect responses in patients with mRCC after only 15 days of sunitinib treatment and to successfully associate these responses with clinical outcome [19] indicating that the therapeutic activity starts early. Our present findings following 14 days of treatment thus confirm these earlier ones. Furthermore, our observations indicate that elevated uptake of FDG in metastatic lesions prior to commencement of treatment correlates with poor prognosis as shown previously [7].

This study has several limitations. The number of examined patients was relatively small, thus a multivariate analysis could not be performed. The first five patients included were examined with older PET equipment (i.e. not with integrated PET/CT) and two patients underwent PET/CT at a different hospital. Although we do not believe that this was likely to influence the outcome. Our ambition was to examine all of the patients on the 14^th^ day of treatment, but this was not always feasible. Four patients did not undergo all three scans for various reasons. Patients were treated with agents with different kinase inhibitory profiles, which could possibly have affected the PET outcome profiles. The agents used in this study are all potent inhibitors of VEGFR-2, but it is not known how this and other various biophysical properties impact on FDG-uptake. All of the patients administered sunitinib had received no prior treatment, whereas most of our patients treated with sorafenib received this as second-line therapy, which might have affected their susceptibility to TKI treatment. Still, in our separate analysis of patients with no previous treatment, there was an association between metabolic response and overall survival. Metabolic response was arbitrary defined as 30% reduction and progression as 30% increase/or new lesion in PET signal. Some patients experienced borderline increase/decrease in PET signal putting them in different response groups despite small differences. Thus, the number of metabolic responders/progressors in the univariate analysis sometimes vary in between groups. We can provide no other reason for this limited concordance. Nonetheless, the PET response for SULpeak, TLG2.5 and TLG50 observed independently, demonstrated a statistically significant association with patient outcome.

It remains somewhat unclear how to define metabolic response of mRCC with PET. The European Organization for Research and Treatment of Cancer (EORTC) [20] defines partial metabolic response as a decline of SUV of more than 25%. This definition does not take into account which SUV value should be analyzed, the size of the region of interest, the optimal cut-off limits for SUV or the number of lesions that should be analyzed. The proposed PERCIST classification is one attempt to establish more robust PET assessment of response and there are other alternatives which include total lesion glycolysis and metabolic volume of the tumor.

## Conclusion

This study indicates that FDG-PET can be used to assess the response of metastatic renal cell cancer to tyrosine kinase inhibitors after only 14 days of treatment. We demonstrate the importance of volumetric PET-response parameters SULpeak, TLG50 and TLG2.5, and propose these parameters as surrogate indicators of PFS and OS for prospective validation in a larger cohort.

## Competing interests

Per Sandström has received research grants for this study as well as honoraria for lectures and participation on advisory boards from Bayer Schering Pharma and Pfizer. Ulrika Harmenberg has received honoraria for lectures and participation on advisory boards from Bayer Schering Pharma and Pfizer.

## Authors’ contributions

JF had access to all data. Acquisition of data: JF, PG, PS, AL, UH and PW. Study concept and design: JF, PG, PS, AL, UH, PW and LB. Analysis and interpretation of data: JF, PG, PS and AU. Writing manuscript: JF and PS. All authors read and approved the final manuscript.

## Pre-publication history

The pre-publication history for this paper can be accessed here:

http://www.biomedcentral.com/1471-2407/14/408/prepub
